# Supplementary material for: Stance detection with BERT embeddings for credibility analysis of information on social media
Source: PeerJ Comput Sci. 2021 Apr 14;7:e467. doi: 10.7717/peerj-cs.467 (PMC8053013; doi:10.7717/peerj-cs.467)
Supplement: Supplemental Information 1 [file peerj-cs-07-467-s001.zip › code_Data/ReadMe.docx]

File name: news-2.csv

Dataset - We are using the G. McIntire. Fake and Real News Dataset, a quite large dataset and a balanced dataset that contains both fake stories and true news.

File name: Stance_Calculation.py

Stance estimates the relative perspective of two pieces of the text concerning a subject, argument, or issue. The stance calculation python code calculates the similarity score between the title of the articles and the text(body of the article). Cosine distance is a good similarity measure as it determines the document similarity. Mathematically, it measures the cosine of the angle between two vectors projected in a multi-dimensional space.

File name: Fake_News_Detection_Stance_BERT.py

The fake news detection system uses the content based information along with the calculated stance values. Our model makes use of BERT embeddings and deep learning algorithms like Recurrent Neural Networks and Convolutional Neural Networks.
